# Hydrometrocolpos in an infant with urological complications: a rare case and review of literature

**DOI:** 10.1093/jscr/rjad406

**Published:** 2023-07-12

**Authors:** Bartholomeo N Ngowi, Frank Bright, Jay Lodhia, Mathias Kimolo, Denis Shirima, Jasper S Mbwambo, Alfred K Mteta, Orgeness J Mbwambo

**Affiliations:** Faculty of Medicine, Kilimanjaro Christian Medical University College, P.O. Box 2240, Moshi, Tanzania; Department of Urology, Kilimanjaro Christian Medical Centre, P.O. Box 3010, Moshi, Tanzania; Faculty of Medicine, Kilimanjaro Christian Medical University College, P.O. Box 2240, Moshi, Tanzania; Department of Urology, Kilimanjaro Christian Medical Centre, P.O. Box 3010, Moshi, Tanzania; Faculty of Medicine, Kilimanjaro Christian Medical University College, P.O. Box 2240, Moshi, Tanzania; Department of General Surgery, Kilimanjaro Christian Medical Centre, P.O. Box 3010, Moshi, Tanzania; Faculty of Medicine, Kilimanjaro Christian Medical University College, P.O. Box 2240, Moshi, Tanzania; Faculty of Medicine, Kilimanjaro Christian Medical University College, P.O. Box 2240, Moshi, Tanzania; Faculty of Medicine, Kilimanjaro Christian Medical University College, P.O. Box 2240, Moshi, Tanzania; Department of Urology, Kilimanjaro Christian Medical Centre, P.O. Box 3010, Moshi, Tanzania; Faculty of Medicine, Kilimanjaro Christian Medical University College, P.O. Box 2240, Moshi, Tanzania; Department of Urology, Kilimanjaro Christian Medical Centre, P.O. Box 3010, Moshi, Tanzania; Faculty of Medicine, Kilimanjaro Christian Medical University College, P.O. Box 2240, Moshi, Tanzania; Department of Urology, Kilimanjaro Christian Medical Centre, P.O. Box 3010, Moshi, Tanzania

## Abstract

Hydrometrocolpos is a rare congenital anomaly characterized by gross distension of the uterus and vagina with fluid, which may result in obstruction to the urine flow. The insertion of Foley catheter into the uterus can relieve the obstruction to the flow of urine and improve renal function. Herein we present a case of infant who was diagnosed with an abdominal mass and renal insufficiency that was managed by the placement of Foley catheter into the dilated uterus and the renal function recovered.

## INTRODUCTION

Hydrometrocolpos is the accumulation of secretions in both the vaginal and uterine cavity. It is a rare condition with an incidence of around 0.006% per year in full-term babies [1, 2]. It is caused by the failure of drainage of cervical/vaginal mucous secretions as a result of distal vaginal obstruction. The condition can be isolated or syndromic [1, 3]. Hydrometrocolpos presents with an abdominal mass and pressure related symptoms due to compression of nearby structures such as urinary bladder, bowel and blood vessels [1, 4]. We present an infant who was diagnosed with an abdominal mass associated with obstructive uropathy that was found to be hydrometrocolpos.

## CASE PRESENTATION

A 4-month-old female infant, with a month history of gradual onset of abdominal distension that was associated with on/off vomiting, intermittent low-grade fevers, crying during both urination and defecation as she passed small amount of urine and hard stool. Other systems, prenatal, natal and postnatal history were unremarkable. She was febrile, temperature 39.9 °C, pale, pulse rate 102 b/min, saturating at 98% in room air and respiratory rate of 38 b/min. On examination of the abdomen, there was a smooth non-tender mass extending to the right upper and left lower quadrants. The mass was non-pulsatile with limited mobility, which could not go below its inferior margin. Bowel sounds were heard normally, and other systems were unremarkable.

Provisional diagnoses of infected large abdominal pelvic teratoma with a differential diagnosis of sascrococygeal teratoma, urachal mass and distended bladder were entertained. The full blood picture revealed haemoglobin of −8.5 g/dl, leukocytosis 16.79 × 10^9^ /L, with neutrophil predominance, platelets were 130 × 10^9^/L^−1^, serum potassium was elevated at 5.62 mmol/L, sodium 137 mmol/L, urea −26 mmol/L, serum creatinine −406 micromoles/L. The urinalysis revealed protein +, leucocytes ++, Blood +++, WBC-10-15/HPF, RBC-1-5/HPF, bacteria +++. The urine culture grew *Escheria Coli that* was sensitive to ceftriaxone, cefepime, ceftazidime, ciprofloxacin, cefotaxime gentamicin, amikacin and meropenem, and resistant to amoxylin/clavulanic acid ampicillin and trimethoprim/sulfamethoxazole.

**Figure 1:**
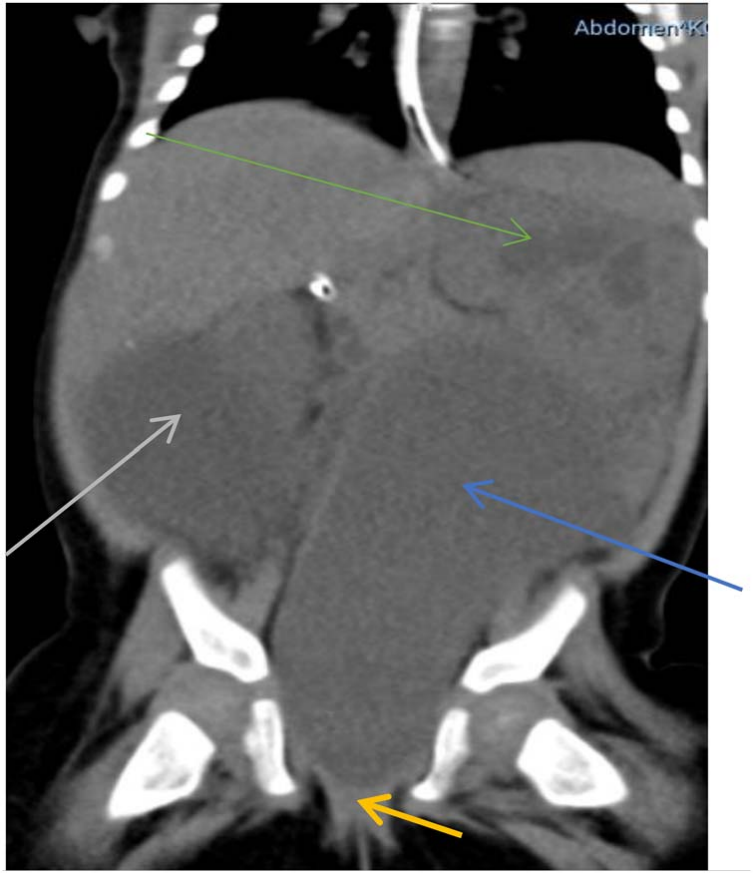
Hypodense mass arising from the uterus (blue arrow), connected to the upper vagina (yellow arrow) pushing the urinary bladder to the right (grey arrow) associated with hydronephrosis (green arrow).

Abdominal X-RAY both supine and erect revealed a soft tissue mass involving the lower quadrant of the abdomen. However, there was no sign of intestinal obstruction. Abdominopelvic ultrasound showed a large, well-defined, anechoic intra-abdominal mass measuring 12 cm × 6 cm, bilateral moderate hydronephrosis and a thick-walled urinary bladder. The uterus could not be appreciated but ovaries were normal. Computed tomography (CT) revealed a centrally located hypodense cystic mass arising from the uterus connected to the upper vagina, located between the urinary bladder and rectum, measuring 7.6 cm × 3.5 cm × 4.6 cm. The mass was extending to the upper abdomen, abutting the rectum and pushing the urinary bladder to the right causing bilateral moderate hydroureteronephrosis (Fig. 1).

**Figure 2:**
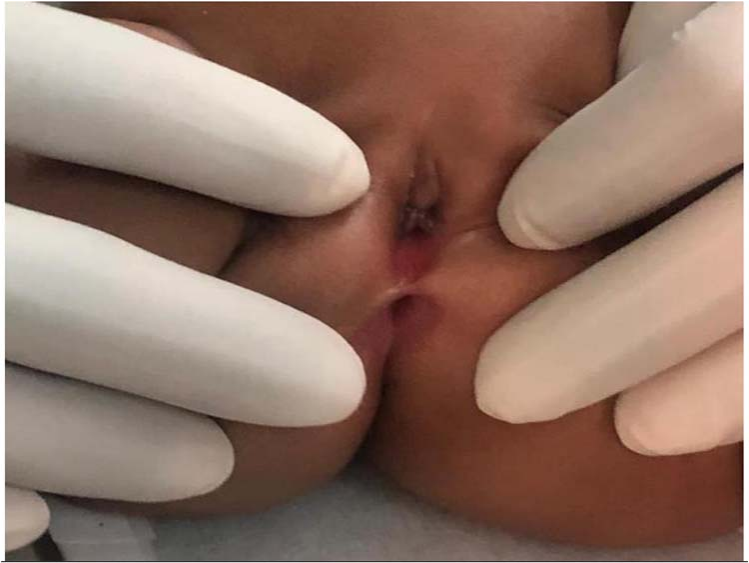
Vagina/introitus separated from the anus by very small septum (blue arrow).

**Figure 3:**
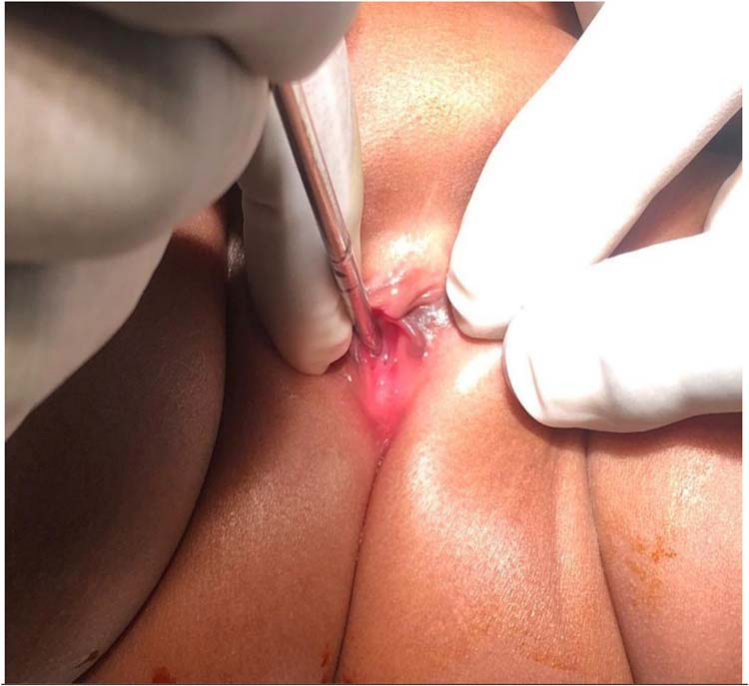
External urethral meatus located more proximal (deep inside introitus) as demonstrated by the metal bogie.

**Figure 4:**
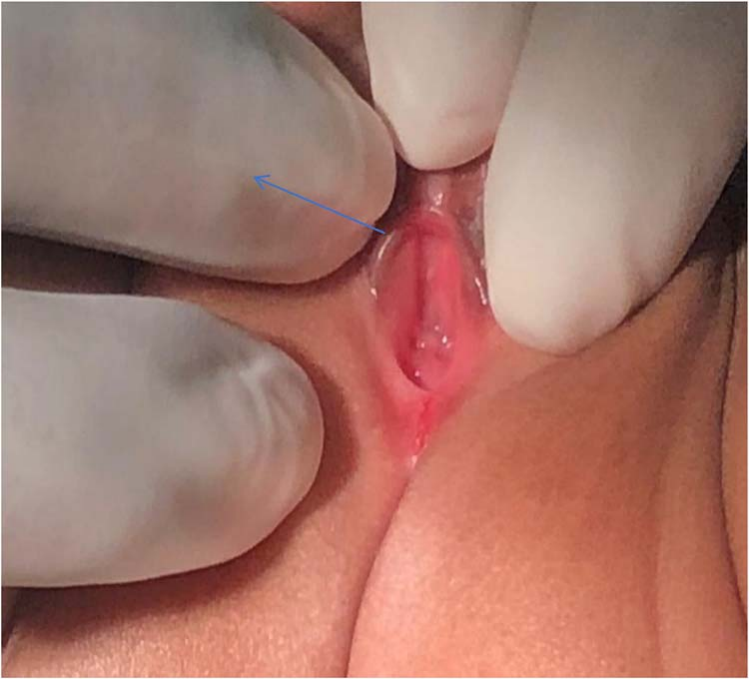
Absence of vaginal opening (blue arrow).

The infant was treated with ceftriaxone for 5 days and fever subsided. A urethral catheter was inserted to monitor urine output and making sure adequate bladder emptying, transfused with 2 units of blood and then planned for cystoscopy and examination under anaesthesia (EUA). During EUA, the anus was displaced more anteriorly, separated from the introitus by a very thin membrane (Fig. 2), the external urethral meatus was located proximal to its normal position (Fig. 3), absent vaginal opening (Fig. 4). Urethrocystoscopy found the ureteric orifices to be displaced to the left, the left ureteric orifice was located between 6 and 5 o’clock, whereas the right orifice was between 5 and 4 o’clock. A 14fr Foley catheter was inserted into the uterus through the anterior abdominal wall, 2 L of turbid fluid was drained. The creatinine dropped to 13 micromoles/L on day 14 post-catheterization of the uterus. Drainage of the genital tract was maintained with a catheter and reconstructive surgery postponed until the child is 2-year old.

## DISCUSSION

Hydrometrocolpos is a rare congenital condition with an incidence of about 0.006% [2–4]. It can be caused by imperforate hymen, transverse vaginal septum, cloacal malformation, distal vaginal agenesis and persistent urogenital sinus, with the former being the most prevalent congenital anomaly [1, 3, 5, 6]. It is classified into five types based on the level of obstruction as well as the severity of the malformation: type I (imperforate hymen), type II (vaginal septum), type III (distal vagina agenesis), type IV (vaginal atresia with persistent urogenital sinus) and the last one is type V, the most severe form that is characterised by atresia of the vagina associated with cloacal malformation [7]. Our case fits in the type III category of hydrometrocolpos.

Hydrometrocolpos can be isolated or associated with syndromes such as the Mckusick Kaufman syndrome, Mayer–Rokitansky–Küste–Hauser syndrome and Herlyn–Werner–Wundelich syndrome [2–4, 7]. Some cases of hydrometrocolpous may have associated abnormal connection with the urinary tract or gastrointestinal tract (fistulae) [5, 8]. Our case did not have any syndromic phenotypic features nor fistula between the urogenital and gastrointestinal tracts.

Hydrometrocolpos is characterised by distension of the vagina and uterus as a result of accumulation of fluid due to distal obstruction presenting as a cystic abdominal mass [2, 5, 7]. There are a number of conditions that presents with cystic mass in children including ovarian cyst, cystic sacrococcygeal teratoma, enteric duplication, urachal cyst and cystic renal masses [1, 2, 8]. It can result into various complications due to pressure effect on the surrounding structures. These include urinary tract obstruction, recurrent urinary tract infection, urine retention (renal insufficiency), rupture of the urinary bladder, sepsis, intestinal obstruction and constipation [2, 4, 5, 7]. Our case presented both constipation and retention of urine due to a severely dilated uterus and vaginal cavity. Some of these complications can be life threatening [1, 5], as this case and hence rapid decompression of the distended vagina/uterus is essential as it is life serving. In this case, we believe the cause of fever was urinary tract infection that was managed by ceftriaxone and bladder drainage using urethral as a catheter.

As definitive reconstructive surgery may be complex and typically postponed until the infant is 2-year old, a temporary solution to relieve the obstructed urinary tract is paramount. The renal function of our patient recovered quickly following placement of the Foley catheter into the distended uterus. A catheter/tube placed into the uterus/vagina is effective in draining the accumulated fluid in the uterus and relieving obstructed urinary tract [4, 5]. The catheters are not without complications, which include dislodgement, causing discomfort or being clogged [5]. In this case, we have not observed any complications so far though she is still under the follow-up.

## CONCLUSION

Hydrometrocolpos is a rare congenital anomaly that should be considered in the differential diagnosis of an abdominal mass in female infant. It can be associated with obstructive uropathy that can be managed by insertion of Foley catheter into the uterus, a simple life-saving procedure.

## CONFLICT OF INTEREST STATEMENT

All listed authors declare that there is no conflict of interest related to publication of this work.

## FUNDING

None.
